# Unique Diagnosis

**Published:** 1870-02

**Authors:** 


### *Unique Diagnosis.*

A FRIEND in DuQuoin sends us the following specimen of professional forces acting at a distance:

DUQUOIN, *Illinois.*

* * Received your letter dated August 22. Inclosed please find two vials of medicine, to be taken, according to direction, during the day. Commence with vial No. 1 and when finished with vial No. 2.

Your disease is a slimy upset on lungs and liver, general prostration of the whole nervous system, some hectic fever, dizziness in the head, pain in the sides and back, not much appetite, and no rest in night-time.

In regard to diet, nothing particular. Don't use strong drinks, as beer, wine or whisky.

Let me know, in fourteen days, what effect these medicines made on you. Respectfully, ***.

### *Portrait of John W. Francis, M.D., LL.D.*

THE publishers of the New York medical and psychological journals have issued a splendid steel engraving of the late eminent and venerated Dr. Francis. It is superb, both as a work of art, and as a "speaking likeness."

There are few names on the roll of honor of the medical profession in this country that can rank with that of Dr. Francis — none surpass. *Nihil tetigit quod non ornavit.* Illustrious in the quiet walks of literature, accomplished as a *savan*, he was the idol of his patients, and the brightest ornament of the social circles in which he moved. The publication of his portrait in such a beautiful style, is a compliment to his memory at once merited and graceful.

It should be hung in the office of every American physician who loves and honors his profession.

### *The Scientific American.*

We welcome this organ and index of the industrial arts and sciences to our exchange list, with the coming in of the new year, and have already marked passages for reproduction in our pages.

This splendidly illustrated weekly journal of popular science, mechanics, invention, engineering, chemistry, architecture, agriculture, and the kindred arts, enters its twenty-fith year on the first of January next, having a circulation far exceeding that of any similar journal now published.

The editorial department of the *Scientific American* is very ably conducted, and some of the most popular writers in this country and Europe are contributors. Every number has sixteen imperial pages, embellished with fine engravings of machinery, new inventions, tools for the workshop, farm and household, engineering works, dwelling houses, public buildings.

A journal of so much intrinsic value, at the low price of $3.00 a year, ought to have, in this thriving country, a million readers.

Whoever reads the *Scientific American* is entertained and instructed, without being bothered with hard words or dry details.

Terms of *Scientific American* — One year $3 00; six months $1.50; four months $1.00. To clubs of ten and upwards, terms $2.50 per annum. Specimen copies sent free. Address the publishers,

Munn & Co., 37 Park Row, New York.

We most cordially indorse the *Scientific American* as filling a space between the professional and general newspaper, with exactly the kind of information which every intelligent professional man desires. We do not know where to look for an equal amount of valuable information in so small a space, and at so low a price.

***The Western Monthly.***

Devoted to literature, biography, and the interests of the West. Published by the Western Monthly company, No. 18, Tribune Building, Chicago. $3.00 per annum in advance.

This magazine has donned a new dress with the new year, and is filled with instructive and entertaining matter. We recognize articles from some of the ablest writers in the West. In its general appearance, size, arrangement, typography, etc., it closely resembles (*too* closely for our Western taste) the *Atlantic Monthly;* but there is a freshness, freedom and vigor about its contents which its heavy prototype at the "Hub" has not exhibited for many years. We take pleasure in recommending it as an especial substitute for the *Atlantic Monthly*, and similar "heavy weight" magazines from the far East.

***The Pharmacist.***

Our enterprising cotemporary commenced the new year under new auspices; Messrs. Ebert & Sargent, pleading the pressure of business cares, retire from its editorial management, in favor Mr. N. Gray Bartlett, who assumes its control. The new editor, well known as a writer and pharmaceutical chemist, will devote his entire time to his new vocation. In extending to him our hearty good wishes, in his new enterprise, we venture to express the opinion that, if he fills the position as well as did his predecessors, the *Pharmacist* will continue to be all that its most exacting readers could ask. W. H.

***The Earth Closet.***

ATTENTION is called to the advertisement of this great improvement, on the second advertising page of this number. In the next JOURNAL will be found a full exposition of the subject.

***To Contributors.***

SEVERAL valuable articles are on file for early publication. To secure prompt appearance, articles should be sent in not less than ten days prior to the date of issue of the ensuing number. A fortnight will be better still.

***New Journals.***

*The American Practitioner*, Louisville, Kentucky, and the *Baltimore Medical Journal*, reached us too late for further notice at present. Both are excellent.

